# Changing genetic profiles of *Plasmodium falciparum* piperaquine resistance in Southeast Asia over 25 years

**DOI:** 10.1128/aac.01117-25

**Published:** 2026-02-17

**Authors:** Suttipat Srisutham, Kanokon Suwannasin, Aungkana Saejeng, Nardlada Khantikul, Rungniran Sugaram, Aung Pyae Phyo, Stephane Proux, Tiengkham Pongvongsa, Chea Nguon, Rupam Tripura, Nguyen Hoang Chau, Dysoley Lek, Nghia Ho Dang Trung, Thomas J. Peto, James J. Callery, Rob W. van der Pluijm, Chanaki Amaratunga, Mehul Dhorda, Lorenz von Seidlein, Mayfong Mayxay, Nguyen Thanh Thuy-Nhien, Paul N. Newton, Nicholas P. J. Day, Elizabeth A. Ashley, Francois H. Nosten, Frank M. Smithuis, Nicholas J. White, Arjen M. Dondorp, Mallika Imwong

**Affiliations:** 1 Department of Clinical Microscopy, Faculty of Allied Health Sciences, Chulalongkorn University 26683, Bangkok, Thailand; 2 Mahidol-Oxford Tropical Medicine Research Unit, Faculty of Tropical Medicine, Mahidol University 26685, Bangkok, Thailand; 3 Division of Vector-borne Diseases, Department of Disease Control, Ministry of Public Health 37694 , Nonthaburi, Thailand; 4 Shoklo Malaria Research Unit, Faculty of Tropical Medicine, Mahidol University 26685, Mae Sot, Thailand; 5 Centre for Tropical Medicine and Global Health, Nuffield Department of Medicine, University of Oxford 6396, Oxford, United Kingdom; 6 Savannakhet Provincial Health Department, Savannakhet, Savannakhet Province, Lao PDR; 7 National Center for Parasitology, Entomology, and Malaria Control 662286, Phnom Penh, Cambodia; 8 Oxford University Clinical Research Unit, Hospital for Tropical Diseases 160913, Ho Chi Minh City, Vietnam; 9 Worldwide Antimalarial Resistance Network, Faculty of Tropical Medicine, Mahidol University, Bangkok, Thailand; 10 Institute of Research and Education Development, University of Health Sciences, Ministry of Health 374369, Vientiane, Lao PDR; 11 Lao-Oxford-Mahosot Hospital-Wellcome Trust Research Unit, Microbiology Laboratory, Mahosot Hospital 251778, Vientiane, Lao PDR; 12 Myanmar Oxford Clinical Research Unit 561007 , Yangon, Myanmar; 13 Department of Molecular Tropical Medicine and Genetics, Faculty of Tropical Medicine, Mahidol University 26685, Bangkok, Thailand; The Children's Hospital of Philadelphia, Philadelphia, Pennsylvania, USA

**Keywords:** molecular surveillance, *pfcrt *mutations, *pfplasmepsin2 *amplification, piperaquine resistance, *Plasmodium falciparum*, Southeast Asia

## Abstract

Piperaquine resistance in *Plasmodium falciparum* has emerged in Southeast Asia and is strongly associated with mutations in the *pfcrt* gene and amplification of *pfplasmepsin2/3*. This study assessed the frequency of *pfcrt* mutations and *pfplasmepsin2/3* gene amplifications across Southeast Asia over 25 years, which is critical for tracking resistant parasites. A total of 898 *P*. *falciparum* isolates collected from Thailand, Myanmar, Cambodia, Laos, and Vietnam between 1995 and 2023 were analyzed for *pfcrt* mutations, *pfplasmepsin2* copy number, and microsatellite variation around the *pfcrt* locus. During the study period, *pfcrt* mutations in Cambodia across the study period showed that H97Y had the highest prevalence at 15%, followed by T93S at 8%, while I218F was common in 24% of specimens in Srisaket and Ubon in Thailand at 24.44%, followed by G353V at 20%, T93S at 17%, F145I at 13%, and H97Y at 4%. In Tak, Thailand, a mutation was found only in 1995 with 40% at I218F and the remaining wild-type. Yala in Thailand and Kayin State in Myanmar remained wild-type. Vietnam showed frequent T93S at 21%. The prevalence of *pfcrt* mutations observed in this study changed throughout the study period. Over 65% of parasites with *pfcrt* mutations at positions 93, 97, 145, 218, 343, and 353 also had *pfplasmepsin2* amplification. Temporal analysis revealed that in Cambodia, *pfplasmepsin2* CNV emerged first, peaking at 88% prevalence in 2015 after the introduction of DHA-piperaquine, with the subsequent increase of *pfcrt* mutations H97Y and G353V. In Eastern Thailand, both *pfcrt* mutations and *pfplasmepsin2* CNV were highly prevalent, exceeding 70% during 2015–2018. In Vietnam, rapid increases in both markers were observed after 2012, reaching peak by 2017. Microsatellite analysis revealed reduced genetic diversity around mutant *pfcrt* alleles, indicating selective sweeps. This study demonstrates that frequencies of *pfcrt* mutations and *pfplasmepsin2* amplification linked to piperaquine resistance have changed over time, highlighting the importance of ongoing genetic monitoring to inform strategies for preserving artemisinin-based combination therapy efficacy.

## INTRODUCTION

Malaria remains a major public health concern worldwide, with an estimated 249 million cases reported in 2022—a slight increase compared with 2021 (1). Effective treatment is critical to malaria elimination efforts. Historically, chloroquine monotherapy played a central role in malaria treatment programs during the 20th century. However, chloroquine resistance (CQR) in *Plasmodium falciparum* was first identified in Southeast Asia in the late 1950s (2) and subsequently spread across Africa by the late 1970s (3). Resistance to piperaquine (PPQ) monotherapy emerged in the 1980s (3), followed by resistance to monotherapy of artemisinin derivatives in 2008 (4).

Dihydroartemisinin–PPQ (DHA–PPQ) is currently recommended by the World Health Organization as a first-line therapy for uncomplicated *P. falciparum* malaria (5). However, DHA-PPQ failures were first reported in Cambodia between 2008 and 2010 (6–9), in Vietnam in 2015 (10), and more recently in northeastern Thailand between 2015 and 2018 (11). In contrast, in Myanmar, two artemisinin-based combination therapies—artemether–lumefantrine and DHA–PPQ—have retained high therapeutic efficacy at three study sites (12).

The *P. falciparum* CQR transporter (*pfcrt*) gene encodes a 424-amino-acid protein featuring 10 predicted transmembrane domains localized to the digestive vacuole membrane (13, 14). Variants of *pfcrt* alleles contain 4 to 10 nonsynonymous mutations (15, 16), with the K76T substitution playing a central role in conferring CQR (17). This substitution removes a positive charge from a predicted substrate-binding site within PfCRT, allowing protonated chloroquine to exit the digestive vacuole along its electrochemical gradient (13). Additional novel amino acid substitutions—T93S, H97L, H97Y, F145I, I218F, M343I, M343L, C350R, G353V, and G367C—have been implicated in piperaquine resistance (18–21).

The global distribution of *pfcrt* mutations has been extensively documented. Two major resistant haplotypes, C72-V73-M74I-N75E-K76T and C72S-V73-M74-N75-K76T, have been widely observed (22, 23). The C72-V73-M74I-N75E-K76T haplotype predominates in Southeast Asia and Africa, whereas the C72S-V73-M74I-N75E-K76T haplotype is more characteristic of South America, Papua New Guinea, and the Philippines (24–26). In contrast, chloroquine-sensitive strains carry the wild-type C72-V73-M74-N75-K76 haplotype.

Several studies have reported changes in the prevalence of *pfcrt* mutations. For example, following the withdrawal of chloroquine pressure in Africa, a decline in drug-resistant *pfcrt* alleles was observed (27). Furthermore, *pfcrt* point mutations associated with piperaquine resistance have been increasingly reported (11, 18). In Cambodia, *pfcrt* mutations were virtually absent in 2012–2013, but by 2016, 95% of isolates harbored such mutations (18). Initially, M343L and G353V were the most common variants, with G353V becoming predominant by 2016, present in 57% of isolates (18). In South America, the PPQ resistance marker C350R was highly prevalent, observed in 83% of isolates in Suriname and 73% in Guyana between 2016 and 2018 (21). Laboratory studies confirmed that parasites carrying the C350R mutation exhibited survival rates above 10% in the piperaquine survival assay, whereas isolates with the wild-type allele were piperaquine-sensitive (21).

Recent genetic cross studies further confirmed the role of *pfcrt* in piperaquine resistance, identifying the G367C mutation as a direct contributor (28). Unlike previously described PPQ-associated mutations that affect the central cavity of the transporter, G367C is located on the digestive vacuole-facing side of transmembrane domain 9. However, the prevalence of this mutation remains low in Southeast Asia (18).

In Africa, these novel *pfcrt* mutations have not yet been widely detected in field isolates. Nonetheless, gene editing experiments demonstrated that the F145I mutation could confer moderate PPQ resistance in an African parasite strain (GB4 from Gabon) (29).

In addition to *pfcrt* mutations, gene amplification of the *P. falciparum plasmepsin 2* (*pfplasmepsin2*) gene has been associated with piperaquine resistance in Cambodia (30, 31). Consequently, *pfplasmepsin2* amplification has been proposed as a molecular marker for tracking PPQ resistance (11, 32, 33). Although *in vitro* evidence quite convincingly shows that certain *pfcrt* mutations confer resistance (18), this has not been clearly demonstrated for *plasmepsin2* amplification due to technical issues with the assay (34). Importantly, the main selection pressure driving recent *P. falciparum* evolution in the region is artemisinin resistance associated with K13 mutations (35). Because ACTs rely on the artemisinin component, K13-mediated resistance has typically emerged before widespread partner drug resistance, such as to piperaquine, develops (11).

The objective of this study was to evaluate the prevalence and patterns of genetic markers associated with piperaquine resistance in *P. falciparum* isolates collected from Cambodia, Thailand, Laos, Vietnam, and Myanmar. Specifically, the study analyzed mutations in the *pfcrt* gene at codons T93S, H97L, H97Y, F145I, I218F, M343I, G353V, and G367C, along with amplifications of the *pfplasmepsin2* gene. Additionally, the study examined the temporal and geographical distribution of these markers, their associations with national antimalarial drug policies, and the relationship between *pfcrt* mutations and *pfplasmepsin2* amplification. Finally, microsatellite analysis surrounding the *pfcrt* locus was performed to detect evidence of selective sweeps, providing insights into the evolutionary dynamics of drug-resistant parasite populations. By presenting comprehensive genetic data from Southeast Asia, this study aims to contribute to a deeper understanding of the evolution of antimalarial drug resistance and to inform ongoing malaria elimination efforts in the region.

## MATERIALS AND METHODS

### Specimen collection

A total of 898 specimens were collected between 1995 and 2023 from 17 study sites across Cambodia, Thailand, Laos, Vietnam, and Myanmar. Specifically, 401 samples were obtained from Battambang, Pailin, Pursat, Rattanakiri, and Stung Treng in Cambodia; 232 samples from Attapeu, Champasak, Salavan, Savannakhet, and Sekong in Laos; 56 samples from Kayin in Myanmar; 107 samples from Srisaket, Tak, Ubon, and Yala in Thailand; and 102 samples from Binh Phuoc and Khanh Hoa in Vietnam.

### DNA extraction and molecular analysis

DNA was extracted from specimens of patients with confirmed *P. falciparum* infection using commercial DNA extraction kits. The *pfcrt* gene was amplified via nested polymerase chain reaction (PCR) under conditions previously described (36). Mutations in the *pfcrt* gene were identified through Sanger sequencing (Macrogen, Korea). Amplification of the *pfplasmepsin2* gene was assessed by real-time PCR following established protocols (30, 37).

The genetic diversity surrounding the *pfcrt* gene was analyzed using a panel of microsatellite markers positioned approximately between −279 and 468 kbon chromosome 7 (File S1). Each microsatellite was amplified by nested PCR using specific primer sets in two rounds. For the first round (Nest 1), a 20-µL reaction containing 10× PCR buffer, 2 mM MgCl₂, dNTPs, 250 µM of each primer, Taq DNA polymerase, and 1 µL of DNA template. Cycling conditions included 30 cycles with an annealing temperature of 50°C. For the second round (Nest 2), a 20-µL reaction with 10× PCR buffer, 2.5 mM MgCl₂, dNTPs, 250 µM of each primer, and Taq DNA polymerase. Thermal cycling involved five cycles with an annealing temperature of 50°C, followed by 25 cycles with an annealing temperature of 45°C.

## RESULTS

### Prevalence of *pfcrt* mutations associated with piperaquine resistance

**Fig 1:**
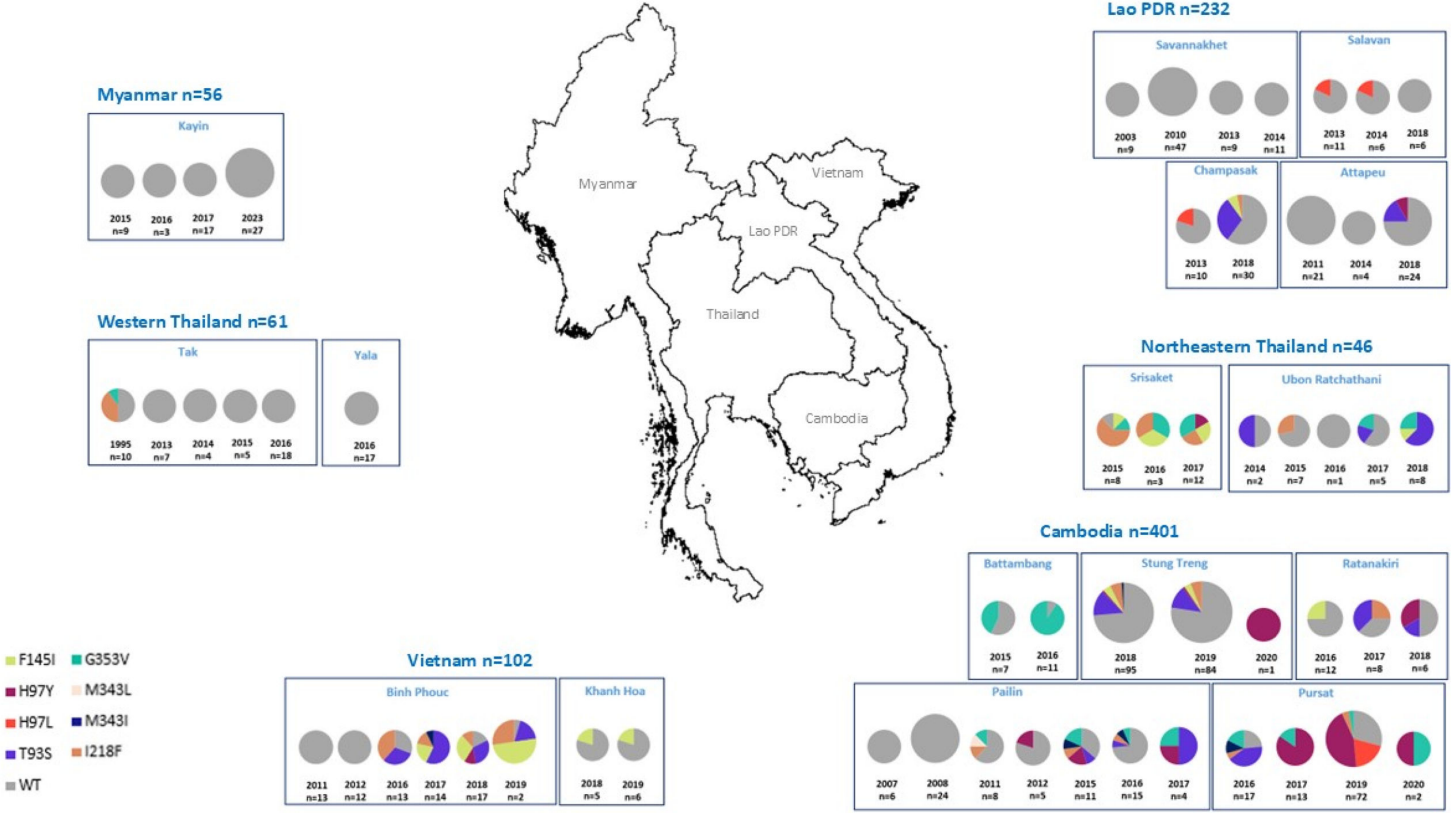
Geographical and temporal distribution of *pfcrt* mutations associated with piperaquine resistance across study sites and countries. The map was created using QGIS.

Among the 898 samples analyzed, *pfcrt* mutations associated with piperaquine resistance were identified as follows. In Pailin, western Cambodia, only wild-type *pfcrt* was detected in 2007–2008 (Fig. 1; File S2 and S3). Mutations first appeared in 2011, with one out of eight (12.5%) isolates each harboring I218F, M343L, and G353V mutations. By 2015–2017, mutations were identified in 30 isolates, including T93S, H97Y, I218F, M343I, and G353V. T93S was observed at 9%, 6%, and 50% prevalence in 2015 (*n* = 11), 2016 (*n* = 15), and 2017 (*n* = 4), respectively. H97Y was observed at 18% in 2015 (*n* = 11) and 25% in 2017 (*n* = 4), while G353V peaked at 18% in 2015 (*n* = 11). I218F and M343I mutations each accounted for 9% in 2015 (*n* = 11). After this period, 2017, no further samples were obtained from Pailin due to the significant reduction in *P. falciparum* cases. In Pursat, also in western Cambodia, where no earlier samples were available, high mutation diversity was observed, particularly in 2019, with H97Y being the most prominent (44.4%; *n* = 72). The H97L mutation was first observed in 2019 (19.4%; *n* = 72), alongside the F145I, I218F, G353V, and M343I mutations. In Stung Treng, northeast Cambodia, 14% and 12% prevalence of the T93S mutation was found in 2018 (*n* = 95) and 2019 (*n* = 84), respectively, along with H97Y, F145I, I218F, and M343I mutations. In Battambang and Rattanakiri, Cambodia, the G353V mutation was detected in Battambang with 43% prevalence in 2015 (*n* = 7) and 91% in 2016 (*n* = 11), while Rattanakiri displayed diverse mutations, including 25% F145I in 2016 (*n* = 12), 38% T93S and 38% I218F in 2017 (*n* = 8), and 33% H97Y and 17% T93S in 2018 (*n* = 6). In Champasak, Laos, the T93S mutation was detected with 30% prevalence in 2018 (*n* = 30), along with 7% F145I and 3% I218F mutations. In Attapeu, Laos, exclusively wild-type alleles were observed until 2018, when T93S and H97Y mutations emerged. In Salavan and Sekong, Laos, the majority of isolates remained wild-type, though some H97L mutations were detected in 2013–2014. In Savannakhet, Laos, all isolates collected in 2003, 2010, 2013, and 2014 were wild-type. In Srisaket, Thailand, a diverse mutation profile was observed in 2015–2017, with approximately 25% each for F145I, I218F, and G353V. In Ubon, Thailand, T93S mutations became prominent in later years, with 63% prevalence in 2018 (*n* = 8), along with 12.5% F145I and 25% G353V. In Tak, Thailand, early mutations were identified in 1995 (*n* = 10), including 40% I218F and 10% G353V, which subsequently was not observed by 2013–2016 (*n* = 34). In Kayin State, Myanmar, all isolates collected from 2015 to 2023 (*n* = 56) remained wild-type. In Binh Phuoc, Vietnam, a shift occurred from predominantly wild-type parasites in 2011–2012 to a diverse mutation profile in 2016–2019. In 2016, the I218F mutation was predominant, found in 38% of 13 isolates. In 2017, 57% of 14 isolates carried the T93S mutation. In 2018 (*n* = 17), 29% had the T93S mutation, equal to the prevalence of F145I, and in 2019, F145I became the most prevalent mutation, detected in 50% of 22 isolates. Additionally, 124 of 898 samples (14%) were screened for the G367C mutation in exon 11 of the *pfcrt* gene using the Sanger sequencing, including 91 samples from Cambodia (2011–2017; *n* = 58), Thailand (2015–2018; *n* = 27), and Vietnam (2016–2017; *n* = 6). All samples were determined to be wild-type.

### Amplification of the *pfplasmepsin2* gene

A total of 851 samples were analyzed for the *pfplasmepsin2* gene copy number variation (CNV). Samples were collected across multiple years from Cambodia (*n* = 385; 2007–2008, 2011–2012, 2015–2020), Laos (*n* = 231; 2003, 2010–2011, 2013–2014, 2017–2018), Thailand (*n* = 93; 1995, 2013–2018), Vietnam (*n* = 101; 2011–2012, 2016–2019), and Myanmar (*n* = 41; 2015, 2017, and 2023).

Parasite isolates from Cambodia exhibited the highest maximum relative quantification (RQ) value of 3.21, indicating the highest *pfplasmepsin2* gene copy number. Vietnam had the second-highest RQ at 2.82, while Thailand exhibited the lowest maximum RQ at 2.55 (File S4).

Multiple copies of the *pfplasmepsin2* gene were identified in 45.97% of samples from Cambodia, and 4.76% of samples from Laos (File S5). In western Cambodia, including Pursat, Battambang, and Pailin, multiple copies of the *pfplasmepsin2* gene were found in 62.78% of samples, compared to 31.22% in the rest of Cambodia. In Thailand, a high prevalence of multiple copies was observed in Ubon (68.81%) and Srisaket (82.61%), both near the Cambodian border. Conversely, in Tak (western Thailand) and Yala (southern Thailand), no isolates carrying multiple *pfplasmepsin2* copies were detected.

In Binh Phuoc, Vietnam, no parasites carried multiple copies in 2011–2012; however, by 2016–2019, 71.21% of parasites exhibited multiple *pfplasmepsin2* gene copies (File S5).

### Association between *pfcrt* mutation and amplification of the *pfplasmepsin2* gene

**Fig 2:**
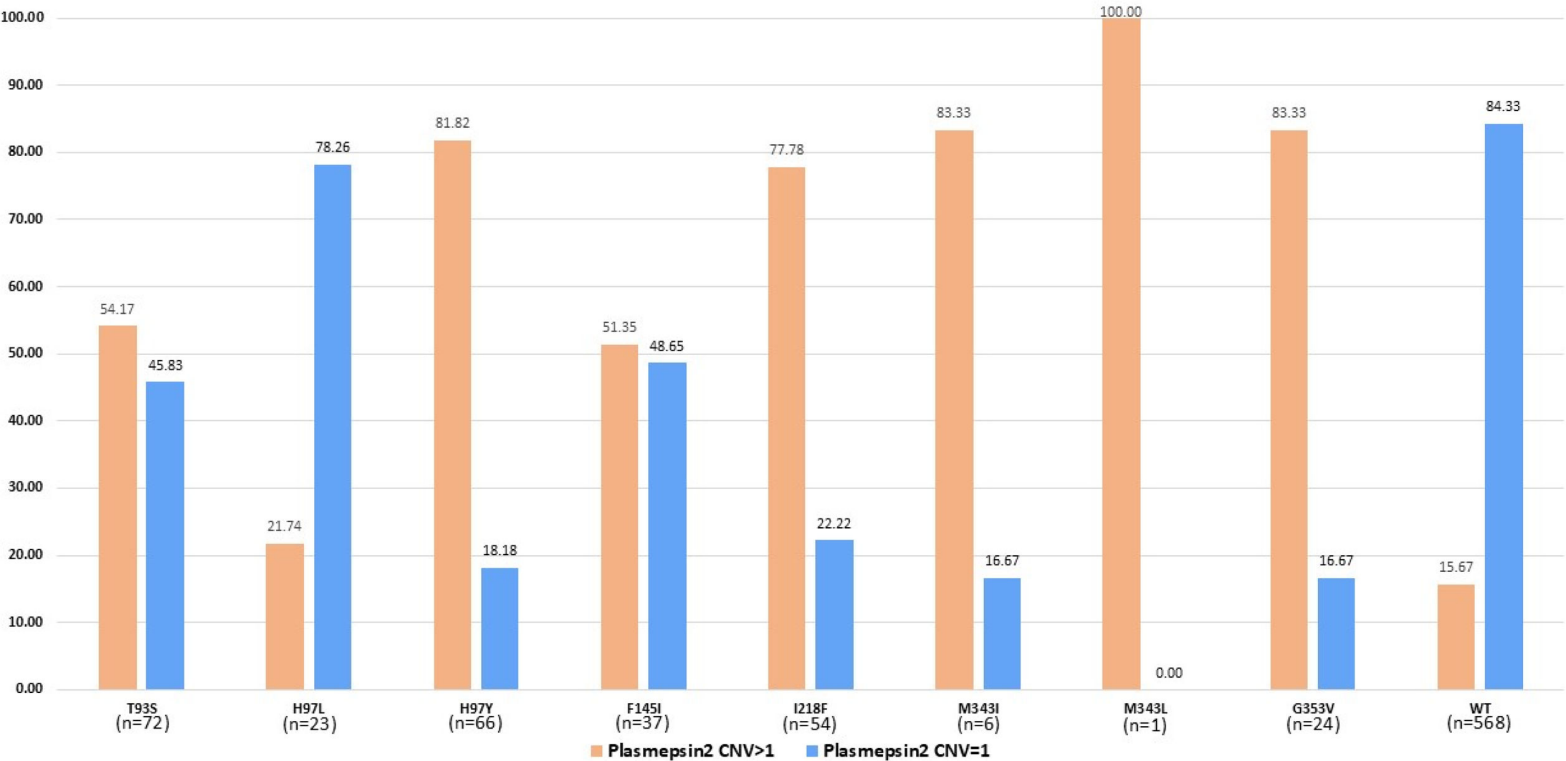
Prevalence of *pfcrt* mutations among samples with single and multiple copies of *pfplasmepsin2*.

**Fig 3:**
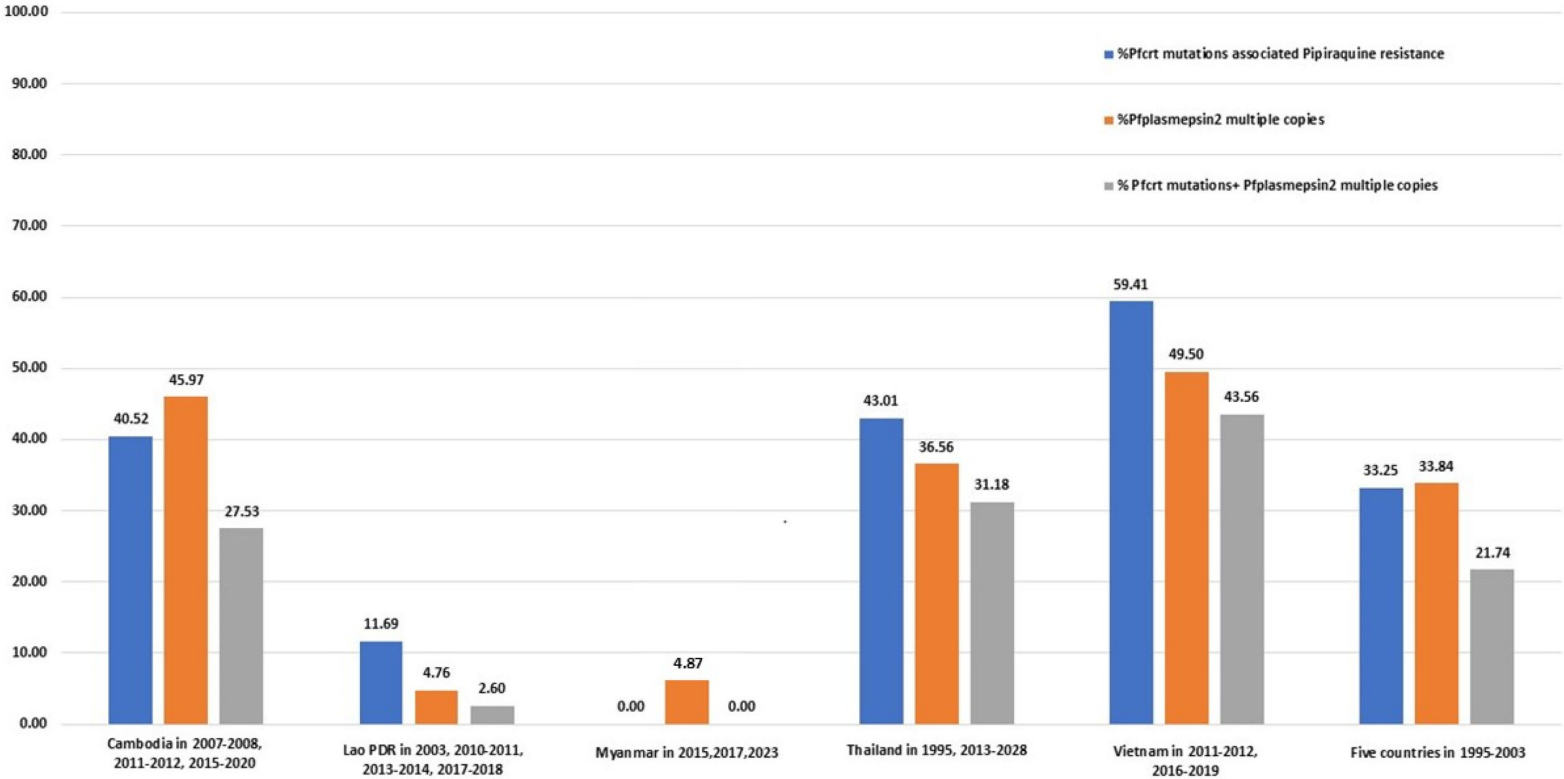
Prevalence of *pfcrt* mutations associated with piperaquine resistance, *pfplasmepsin2* multiple copies, and their co-occurrence across five countries.

A total of 851 samples were successfully analyzed for both *pfplasmepsin2* CNV and *pfcrt* mutations associated with piperaquine resistance. The results demonstrated that over 75% of parasites harboring *pfcrt* mutations (H97Y, I218F, M343I, M343L, and G353V) carried multiple copies of *pfplasmepsin2* (Fig. 2), whereas 22%–54% of parasites harboring T93S, H97L, and F145I carried multiple copies of *pfplasmepsin2*. In Cambodia, the proportion of isolates with *pfcrt* mutations was approximately 41%, while *pfplasmepsin2* multiple copies were observed in about 46%, and the co-occurrence of both markers was seen in 27% (Fig. 3). In Lao PDR, the prevalence was much lower, with *pfcrt* mutations at 12%, *pfplasmepsin2* multiple copies at 5%, and co-occurrence at 3%. In Myanmar, only *pfplasmepsin2* multiple copies were observed, at 39%. In Thailand, *pfcrt* mutations were present in 43% of isolates, *pfplasmepsin2* multiple copies in 36%, and both markers together in 31%. In Vietnam, there were the highest prevalence rates, with 59% for *pfcrt* mutations, 50% for *pfplasmepsin2* multiple copies, and 44% for the co-occurrence of both markers.

In Pailin, Cambodia, a diverse range of *pfcrt* mutations (G353V, H97Y, and I218F) was observed, frequently associated with multiple *pfplasmepsin2* copies (File S5). In Pursat, a high prevalence of the H97Y mutation was noted, often linked to multiple *pfplasmepsin2* copies. In Binh Phuoc, Vietnam, a shift was observed from predominantly wild-type *pfcrt* and a single *pfplasmepsin2* copy in 2011*–*2012 to diverse *pfcrt* mutations, particularly F145I, combined with multiple *pfplasmepsin2* copies by 2019. In Srisaket and Ubon, Thailand, an increase in *pfcrt* mutations (F145I, I218F) and multiple *pfplasmepsin2* copies was recorded from 2015 onward. In Laos and Myanmar, wild-type *pfcrt* and single-copy *pfplasmepsin2* were generally maintained, with few exceptions.

**Fig 4:**
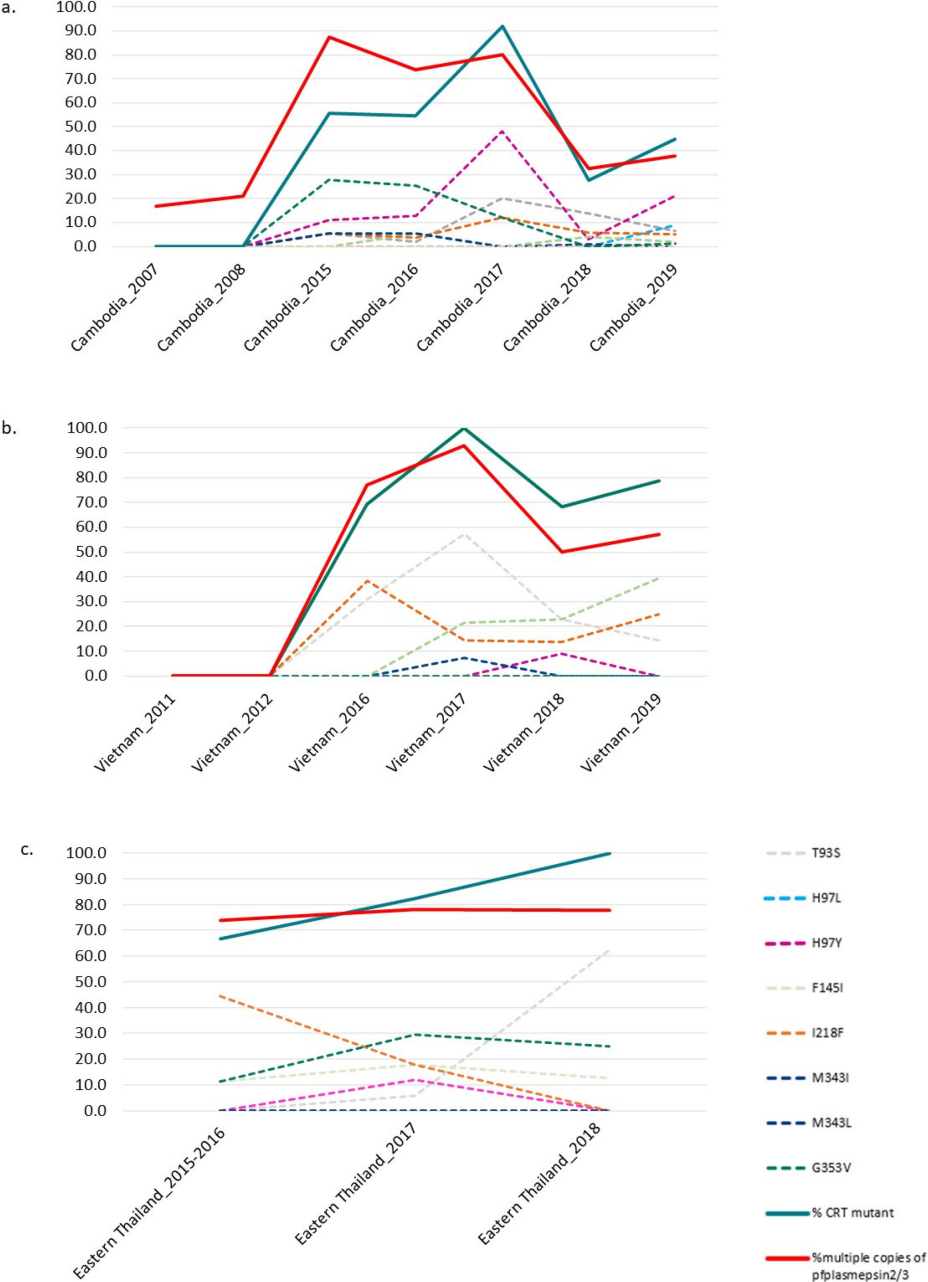
Temporal evolution of *pfcrt* mutations in Southeast Asian *P. falciparum* populations (2007–2019): (**a**) Cambodia, (**b**) Vietnam, and (**c**) eastern Thailand.

Temporal analysis revealed distinct patterns of *pfcrt* mutations and *pfplasmepsin2* CNV across Cambodia, Vietnam, and Thailand. In Cambodia (2007–2019), *pfplasmepsin2* CNV initially emerged as the primary marker of piperaquine resistance, peaking at 88% prevalence in 2015 (Fig. 4a). Subsequently, novel *pfcrt* mutations, particularly H97Y and G353V, emerged. The prevalence of H97Y increased dramatically from 2008, peaking at approximately 50% in 2017, before declining notably in 2018–2019, coinciding with a policy change from dihydroartemisinin–piperaquine to artesunate–mefloquine. The prevalence of *pfplasmepsin2* CNV similarly declined to 33%–38% by 2018–2019.

Vietnam exhibited parallel trends, with both *pfcrt* mutations and *pfplasmepsin2* CNV emerging rapidly after 2012, reaching peak frequencies of approximately 93% and 100%, respectively, by 2017 (Fig. 4b). Various *pfcrt* point mutations (T93S, H97Y, F145I, I218F, and M343I) showed distinct temporal patterns, with T93S being the most prevalent. Other markers such as H97Y, F145I, I218F, and M343I remained at frequencies below 40%.

In Eastern Thailand (2015–2018), *pfcrt* mutant prevalence increased sharply from approximately 65% to nearly 100% (Fig. 4c). *pfplasmepsin2* multiple copies remained consistently high (approximately 75%–80%). Notably, I218F declined substantially from approximately 45% to nearly 0, while G353V initially increased but stabilized around 30%. Less prevalent mutations (H97L, H97Y, and F145I) fluctuated modestly, generally staying below 15%. T93S emerged later, showing an increasing trend toward 2018.

These findings highlight a dynamic evolutionary landscape for genetic markers of drug resistance, likely reflecting selective pressure from antimalarial drug use.

### Microsatellite marker analysis

**Fig 5:**
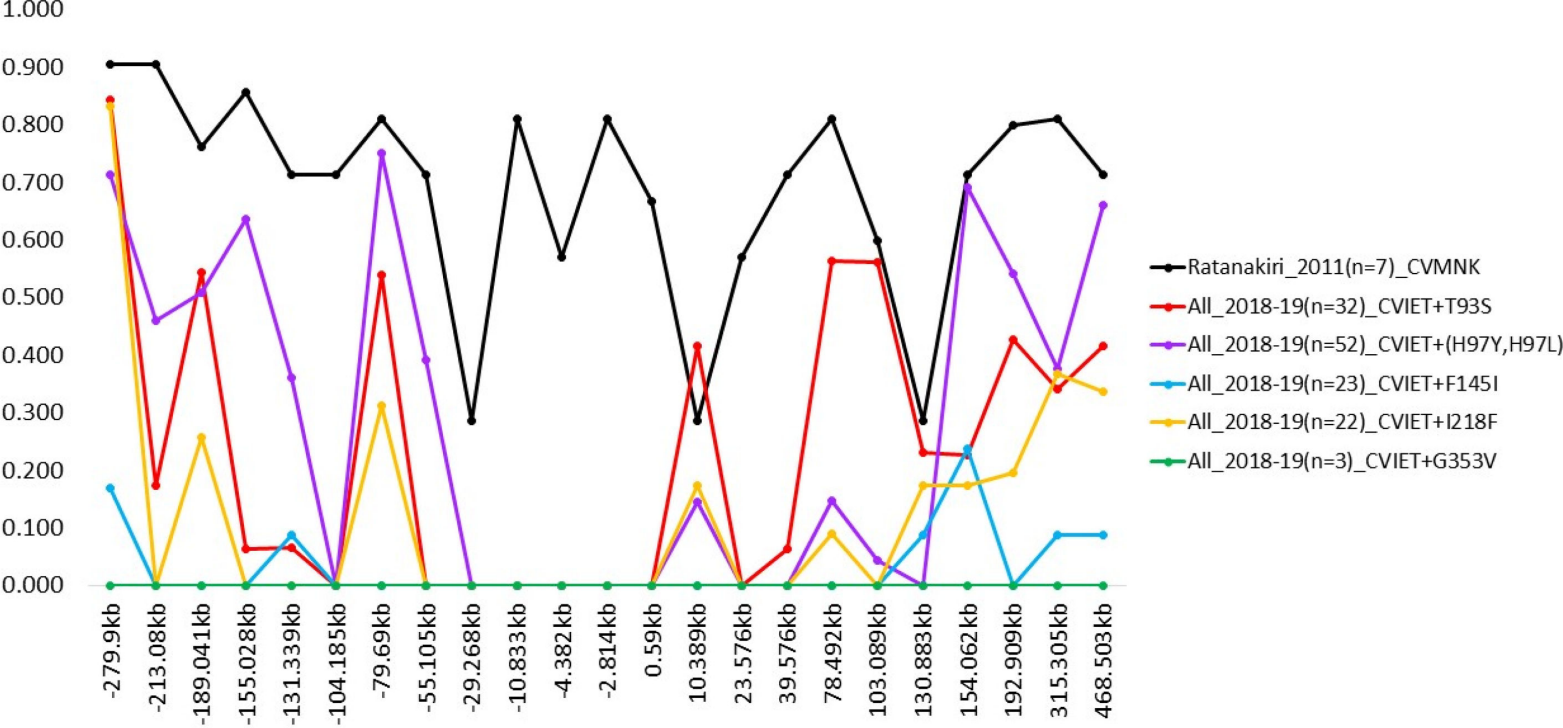
Microsatellite marker analysis of genetic diversity surrounding the *pfcrt* gene on chromosome 7. The graph displays multiple data series comparing different genetic variants (C72-V73-M74I-N75E-K76T+T93S, C72-V73-M74I-N75E-K76T+(H97Y, H97L), C72-V73-M74I-N75E-K76T+F145I, C72-V73-M74I-N75E-K76T+I218F, and C72-V73-M74I-N75E-K76T+G353V) against reference isolates from Ratanakiri in 2011. The *x*-axis shows chromosomal positions in kilobases (kb), spanning approximately 748.403 kb, and the *y*-axis represents genetic diversity values ranging from 0 to 1.0. Data points are connected by lines to show patterns of diversity across the chromosomal region.

The genetic diversity surrounding the *pfcrt* gene was assessed using a panel of microsatellite markers spanning a total of 748.403 kb on chromosome 7. The control specimens of *P. falciparum* parasite isolates from Ratanakiri, Cambodia (2011), carrying the CVMNK haplotype, exhibited high genetic diversity (0.8–0.9) throughout the examined region (Fig. 5). In contrast, parasite isolates from Cambodia and Vietnam (2018–2019) with the CVIET variant showed markedly reduced diversity, particularly near the *pfcrt* locus.

Parasites with haplotypes CVIET+T93S (*n* = 32) and CVIET+(H97Y, H97L; *n* = 52) exhibited similar patterns, with a lack of diversity between −29.268 kb and 0.59 kb, indicating potential selective sweeps. More pronounced reductions were observed in parasites with the CVIET+F145I (*n* = 23), +I218F (*n* = 22), and +G353V (*n* = 3) haplotypes, where diversity values were zero, especially in proximal regions. These results suggest strong selective pressure on these drug resistance-associated haplotypes, consistent with recent selective sweeps in parasite populations.

### Prevalence of *pfcrt* mutations associated with CQR

**Fig 6:**
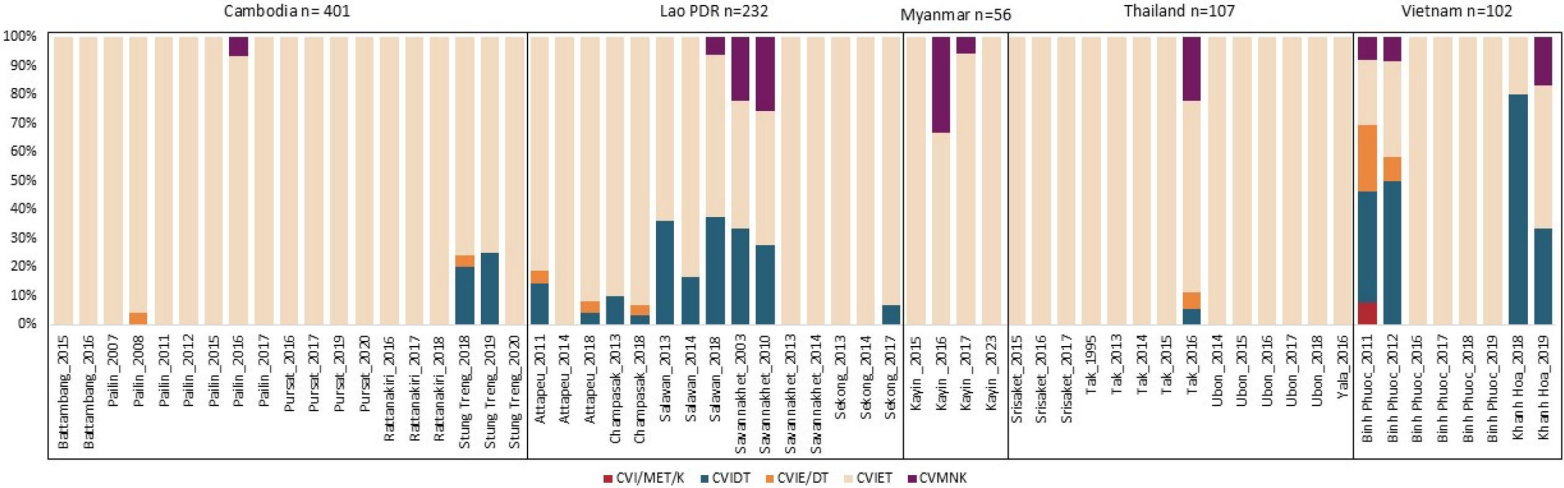
Prevalence of *pfcrt* haplotypes associated with chloroquine resistance.

Among 898 samples from five countries, four *pfcrt* haplotypes (codons 72–76) were identified: C72-V73-M74-N75-K76 (wild-type), 2.78%; C72-V73-M74I-N75E-K76T (mutant type), 85.41%; C72-V73-M74I-N75D-K76T (mutant type), 10.24%; and mixed types (C72-V73-M74M/I-N75E-K76K/T and C72-V73-M74I-N75E/D-K76T), 1.56%. The CVIET haplotype was predominant across study sites in Thailand, Lao PDR, Cambodia, Myanmar, and Vietnam (Fig. 6; File S6). In Cambodia, CVIET was consistently dominant. In Pailin, this haplotype persisted from 2007 to 2017, but then no more samples were collected as *P. falciparum* was effectively eliminated. In Stung Treng, a shift from CVIDT in 2018–2019 to 100% CVIET in 2020 was observed. In Lao PDR, greater haplotype diversity was noted compared to Cambodia, with both CVIET and CVIDT observed. Notably, CVMNK (wild-type) was still present in earlier years (2003, 2010) in Savannakhet. In Myanmar, in Kayin state (2015–2023), CVIET was the primary haplotype, with occasional observations of wild-type parasites (2016–2017). In Thailand, CVIET was most common across sites although some haplotype diversity was noted in Tak Province in 2016. In Vietnam, in Binh Phuoc Province, a shift from mixed haplotypes in 2011–2012 to CVIET dominance by 2016 was recorded. Khanh Hoa Province showed the presence of CVIDT, CVIET, and CVMNK haplotypes.

## DISCUSSION

Artemisinin combination therapy (ACT) has been used as the first-line treatment for uncomplicated *P. falciparum* infections in Southeast Asia (38). Between 2002 and 2004, reports of artesunate–mefloquine (AS/MQ) treatment failures emerged in Pailin, western Cambodia, and Trat, Thailand (39, 40). Due to rising AS/MQ failure rates, western Cambodia adopted DHA/PPQ as first-line therapy in 2008 (41, 42). However, DHA/PPQ failures were soon reported, leading to a reversion to AS/MQ in regions with significant DHA/PPQ failures (7).

In this study, we reported the prevalence of *pfcrt* mutations, including H97Y, T93S, and I218F, between 2011 and 2020, during the period when DHA/PPQ was the first-line treatment in Cambodia. Baseline samples collected from Pailin, Cambodia, during 2007–2008, when AS/MQ was still the first-line therapy, showed no *pfcrt* mutations at T93S, H97L/Y, F145I, I218F, M343I, and G353V. Additionally, *pfplasmepsin2* gene amplification increased in Pailin from 20% in 2007–2008 to 100% by 2011–2015 following the switch from AS/MQ to DHA/PPQ. These findings suggest that both *pfcrt* mutations and *pfplasmepsin2* gene amplification contribute to piperaquine resistance.

The temporal evolution of *pfcrt* mutations and *pfplasmepsin2* copy number changes in Cambodia from 2007 to 2019 revealed significant genetic shifts. In 2007, *pfcrt* mutations were rare, and only about 10% of parasites had multiple copies of *pfplasmepsin2*. However, after the 2008 introduction of DHA/PPQ, both *pfcrt* mutations and *pfplasmepsin2* amplification rose dramatically. In Cambodia, CVIET was consistently dominant, but in Stung Treng, there was a shift from CVIDT in 2018–2019 to 100% CVIET in 2020. This shift occurred after Artesunate–Mefloquine (ASMQ) had been used in this area since 2017. Additionally, Artemether–Lumefantrine (AL) and the triple combination Artemether–Lumefantrine–Amodiaquine (ALAQ) were introduced as study drugs from the start of the TACTCV study in 2018. The prevalence of *pfplasmepsin2* amplification peaked at nearly 90% in 2015, while total *pfcrt* mutations reached about 90% in 2017. Notably, the H97Y mutation emerged strongly after 2016 and peaked around 2017, and G353V maintained a steady presence in later years. When compared with a recent study, which reported that the proportion of parasite isolates from Cambodia carrying the H97Y mutation was highest in 2020 and then declined in 2021 and 2022 (43), our findings show a similar pattern. In our study, the prevalence of H97Y decreased but appeared to increase again in 2019, and the study in 2020 also reported a higher prevalence. After 2017, both *pfcrt* mutations and *pfplasmepsin2* amplification declined markedly, stabilizing at approximately 40% by 2019, coinciding with Cambodia’s gradual return to AS/MQ treatment between 2014 and 2017 (32). According to recent studies, the frequency of pfplasmepsin2 amplifications decreased dramatically from 62% in 2017–2019 to just 2% in 2022, coinciding with a shift in frontline malaria therapy in Cambodia, Thailand, and Vietnam (43). From the results of this study, the findings were also consistent with previous research conducted in Northern Cambodia (44), which reported the emergence of the *pfcrt* H97Y mutation beginning in 2014, with its prevalence increasing through 2017. The *pfcrt* F145I mutation rapidly increased in prevalence from 2010 to 2014, while the G353V mutation was not observed until 2017. In addition, a decline in the prevalence of *pfplasmepsin2* amplification was observed in 2017 compared to 2015 in Northern Cambodia, which is also consistent with the findings of this study. The H97Y, F145I, M343L, and G353V mutations were previously clinically confirmed to confer resistance to PPQ (18). Although previous studies have found that the *in vitro* data do not explain selection of the genotypes under field conditions, changes in piperaquine survival assay (PSA) results and related in-vitro phenotypes have been observed as dihydroartemisinin–piperaquine (DP) use became more widespread (30, 45). This study revealed a key temporal pattern that *pfplasmepsin2* amplification emerged earlier than *pfcrt* mutations in Cambodian parasite populations. Nevertheless, we propose *pfplasmepsin2* amplification facilitates the selection of *pfcrt* mutations. However, the *pfcrt* mutations also occur in the absence of *plasmepsin2* amplification. Among parasite population with *pfcrt* mutations that retained long haplotype (as a measure of selection), 68% also harbored *pfplasmepsin2* amplification. This co-occurrence suggests that the combination of *pfcrt* mutations and *pfplasmepsin2* amplification may confer higher levels of PPQ resistance; further functional studies are needed to confirm this association.

In northeastern Thailand (Srisaket and Ubon Ratchathani), resistance to DHA/PPQ was reported with day 42 efficacy rates of 75.9% in FY2018 and 49.4% in FY2019, respectively (46). Between 2014 and 2018, we detected both *pfcrt* mutations associated with piperaquine resistance and *pfplasmepsin2* gene amplification. The prevalence of *pfcrt* mutations increased from approximately 65% to nearly 100%, while *pfplasmepsin2* amplification remained relatively stable at 75%–80%. During 2015–2016, *pfplasmepsin2* amplification was more prevalent than *pfcrt* mutations, but by 2018, *pfcrt* mutations had become the dominant marker. The high prevalence of both markers suggests enhanced piperaquine resistance and potentially reduced drug efficacy. Consequently, first-line treatment in eastern Thailand shifted from DHA/PPQ to artesunate–pyronaridine (11, 47). However, molecular markers for pyronaridine resistance have not yet been validated (48), and DHA/PPQ continues to be used in most areas of Thailand except in the northeast (47). In this study, no *pfcrt* mutations or *pfplasmepsin2* amplifications were detected in Yala province, indicating high piperaquine efficacy in these regions. In Tak Province, I218F and G353V mutations were found in specimen isolates from 1995, whereas no mutations were detected in specimen isolates collected during 2013–2016. Moreover, *pfplasmepsin2* amplifications were not detected in southern Thailand, whereas they were observed in northeastern Thailand. Therefore, the molecular marker distribution across Thailand’s regions demonstrates varying effectiveness of antimalarial drugs in each area, highlighting the need for close monitoring of parasite populations. In Myanmar, various antimalarials, including chloroquine (CQ), sulfadoxine–pyrimethamine (SP), and mefloquine (MFQ), were used around 2000 (49). Currently, ACTs such as artemether–lumefantrine, artesunate–mefloquine, and DHA/PPQ are employed as first-line treatments (50). We analyzed parasite isolates from Kayin, Myanmar (2015, 2017, and 2023). Parasites carried the *pfcrt* CVIET haplotype, without mutations at T93S, H97L/Y, F145I, I218F, M343I, and G353V. Previous studies conducted in the China–Myanmar region in 2007 and 2010 and in the Thailand–Myanmar region in 2012 and 2014 also reported similar results, showing no new *pfcrt* mutations conferring resistance to PPQ, such as T93S, H97Y, F145I, M343L, and G353V. Only the mutant I218F was identified, with a prevalence of 2.27% along the Thailand–Myanmar border (51).

In Lao PDR, artemether–lumefantrine (CoArtem) has been the first-line treatment since 2004 (52), with DHA/PPQ used in some areas (53). At that time, studies demonstrated that dihydroartemisinin–piperaquine did not have superior efficacy compared to artesunate–mefloquine for the treatment of uncomplicated falciparum malaria in Laos in 2004 (50). In this study, isolates collected in 2003, 2010, and 2011 showed no *pfcrt* mutations at positions T93S, H97L/Y, F145I, I218F, M343I, G353V, and G367C, and also had a single copy of pfplasmepsin2, which is consistent with the drug efficacy observed during that period. Nevertheless, a few parasites carried H97L, H97Y, T93S, I218F, and F145I mutations. *Pfplasmepsin2* gene amplification was detected in 5% of the specimens isolated from Lao PDR (*n* = 231) in Attapeu, Champasak, and Salavan provinces in 2018. This may be due to continued antimalarial drug use in the region, leading to drug selection pressure or possible migration of parasites. In Vietnam, DHA/PPQ has been the first-line treatment for uncomplicated *P. falciparum* since 2007 (54). After confirming piperaquine resistance (55), artesunate–pyronaridine was recommended in some areas, such as Binh Phuoc (56). No *pfcrt* mutations were observed in Binh Phuoc isolates from 2011 to 2012. However, between 2016 and 2019, mutations including T93S, I218F, F145I, H97Y, and M343I, along with *pfplasmepsin2* amplification, increased. This finding [ss1] is consistent with a study conducted between 2018 and 2022 in Vietnam, which also reported the presence of the T93S and I218F mutations (43). This concurrent rise indicates growing piperaquine resistance and the potential for multidrug resistance (11, 57, 58). A previous study also reported high prevalence of PPQ resistance markers T93S, H97Y, and F145I in the *pfcrt* gene in Binh Phuoc and Binh Thuan provinces, Vietnam (63% and 84%, respectively) (59). These genetic trends correlate with rising clinical treatment failures in regions like Binh Phuoc, where failure rates have reached concerning levels (11).

Recent studies suggest *pfcrt* mutations contribute significantly to piperaquine resistance. A genetic cross study (28) showed that the G367C mutation in *pfcrt* confers resistance via the digestive vacuole-facing domain of transmembrane helix 9, rather than the transporter’s central cavity. Previous studies demonstrated that G367C is rare in Southeast Asia (28), which is consistent with our findings that the absence of the G367C mutation in our samples indicates limited or no selection pressure for this particular mutation in the studied populations. Moreover, previous research demonstrates that piperaquine-resistant *pfcrt* mutations in *P. falciparum* differentially impact parasite physiology during the blood stage by altering drug transport, hemoglobin digestion, and overall cellular metabolism (60). These mutations reduce intracellular accumulation of piperaquine and change hemoglobin catabolism, leading to increased hemoglobin-derived peptides and potential effects on parasite fitness (20). The amplification of *pfplasmepsin2* in piperaquine (PPQ) resistance involves enhanced hemoglobin digestion in *P. falciparum* parasites. Amplification of these genes—which encode hemoglobin-degrading proteases in the digestive vacuole—is hypothesized to accelerate hemoglobin breakdown, reducing toxic heme accumulation, a key target of PPQ’s disruption of heme detoxification (30). Continued monitoring of *pfcrt* mutations and *pfplasmepsin2* amplification is important not only to track their prevalence but also to understand how these variants evolve, shift, and interact under drug pressure. Microsatellite analysis revealed distinct genetic diversity patterns surrounding the *pfcrt* gene. Wild-type C72-V73-M74-N75-K76 parasites from Ratanakiri in 2011 exhibited high diversity (0.8–0.9), indicating natural variation before drug pressure (61). In contrast, parasites collected during 2018–2019 bearing C72-V73-M74I-N75E-K76T variants showed reduced diversity, particularly in flanking regions of the *pfcrt* locus. Parasites with C72-V73-M74I-N75E-K76T+F145I, +I218F, and +G353V variants exhibited the most pronounced reductions, suggesting selective sweeps (62). These patterns imply a temporal sequence of resistance emergence, with some mutations reflecting more recent adaptations. Similar sweeps have been observed in chloroquine-resistant parasites (63).

Compared with isolates from Africa, the *pfcrt* mutations observed here differ significantly. Piperaquine resistance-associated *pfcrt* mutations remain rare in African *P. falciparum* populations. A molecular surveillance study of African isolates from 2017 to 2018 found no evidence of key mutations like T93S, H97Y, F145I, M343L, C350R, and G353V, which are common in Southeast Asia (64). In addition, a recent study showed that *P. falciparum* African *pfcrt* haplotypes carrying Southeast Asian-type mutations such as T93S or I218F did not confer piperaquine resistance, and that these mutations impose a major fitness cost, which may limit their spread (65).

This study provides genetic data from five Southeast Asian countries where ACT has been used as the first-line treatment. Piperaquine was mainly the partner drug. Our analysis shows that *pfcrt* mutations (T93S, H97L/Y, F145I, I218F, M343I, G353V, and G367C) and *pfplasmepsin2* gene amplification are prevalent in regions where piperaquine was used, while baseline samples from piperaquine-free areas lacked these markers. Close monitoring of molecular markers remains critical to support malaria elimination efforts.

## Data Availability

All data generated or analyzed during this study are included in this published article and its supplemental material.

## SUPPLEMENTAL MATERIAL

File S1 aac.01117-25-s0001.pdf Primers used for analysis of microsatellites in the flanking region of the *Pfcrt* gene.

File S2 aac.01117-25-s0002.pdf Prevalence of *Pfcrt* mutations associated with piperaquine resistance across study sites and countries by year.

File S3 aac.01117-25-s0003.xlsx PfCRT haplotypes at positions 72, 73, 74, 75, and 76 and PfCRT mutations at positions 93, 97, 145, 218, 343, and 353.

File S4 aac.01117-25-s0004.pdf Copy number variation (CNV) of *pfplasmepsin2* in Cambodia, Thailand, and Vietnam.

File S5 aac.01117-25-s0005.xlsx Pfcrt mutations associated with piperaquine resistance and pfplasmepsin2 gene copy number variations.

File S6 aac.01117-25-s0006.pdf Prevalence of *Pfcrt* haplotypes associated with chloroquine resistance.

ASM does not own the copyrights to Supplemental Material that may be linked to, or accessed through, an article. The authors have granted ASM a non-exclusive, world-wide license to publish the Supplemental Material files. Please contact the corresponding author directly for reuse.
