# Supplementary material for: Changing genetic profiles of Plasmodium falciparum piperaquine resistance in Southeast Asia over 25 years
Source: Antimicrob Agents Chemother. 2026 Feb 17;70(4):e01117-25. doi: 10.1128/aac.01117-25 (PMC13041312; doi:10.1128/aac.01117-25)
Supplement: File S1 — Primers used for analysis of microsatellites in the flanking region of the Pfcrt gene. [file aac.01117-25-s0001.pdf]

Supplementary Table: Primers used for analysis of microsatellites in the flanking region

Marker	Uesd for	Primer name	Primer sequencing	PCR Product (bp)
-279.9kb	Nest 1	CRT -279.9kb_F1	AATGTATCTATCCATTTTATACTT	232
	Nest 1,2	CRT -279.9kb_R	AATAATTTTGTTAACACATCG	
	Nest 2	CRT -279.9kb_F2	HEX-TACATATTTTTGGTTTTTCC	
-213.08kb	Nest 1,2	CRT -213.08kb_F	AGTATTAATTATCACTATCCAATT	136
	Nest 1	CRT -213.08kb_R1	CATATGATAAAGAAGTCGTTC	
	Nest 2	CRT -213.08kb_R2	6FAM-GATAAAGAAGTCGTTCATGTT	
-189.041kb	Nest 1	CRT -189.041kb_F1	GTACATCTTATGGAAGAAGC	172
	Nest 1,2	CRT -189.041kb_R	TTTATAAGGCACACATGAAT	
	Nest 2	CRT -189.041kb_F2	6FAM-GGAAGAAGCCTTAGATAAAA	
-155.028kb	Nest 1,2	CRT -155.028kb_F	ATTTATCTATCGCCTTTGTT	201
	Nest 1	CRT -155.028kb_R1	ATGTGTGATAAATCATCTGG	
	Nest 2	CRT -155.028kb_R2	HEX-TGATAAATCATCTGGAACAT	
-131.339kb	Nest 1	CRT -131.339kb_F1	CTCTTAAAATTGTCCAAACA	178
	Nest 1,2	CRT -131.339kb_R	TGATAGGATAAGTTTTTGAA	
	Nest 2	CRT -131.339kb_F2	HEX-AATTGTCCAAACAAATAAAA	
-104.185kb	Nest 1	CRT -104.185kb_F1	TTATTTAAGATGACTTTTGTAAG	129
	Nest 1,2	CRT -104.185kb_R	GGATTTATAATATTCAAAAGGA	
	Nest 2	CRT -104.185kb_F2	6FAM-AAAAATATAAATTCATGTGC	
-79.69kb	Nest 1	CRT -79.69kb_F1	CATTGATCCATTTATGATT	158
	Nest 1,2	CRT -79.69kb_R	AAAAGCTGTCAATTTTTAATCTA	
	Nest 2	CRT -79.69kb_F2	6FAM-ATTTGTTTTTATTTTTCACC	
-55.105kb	Nest 1	CRT -55.105_F1	AAGACCGTTTAGTTTTAGAAT	145
	Nest 1,2	CRT -55.105_R	TTTTCATTATGTATACGTGTGT	
	Nest 2	CRT -55.105_F2	FAM-CGTTTAGTTTTAGAATGCA	
-29.268kb	Nest 1	CRT -29.268_F1	TATGCATATTCCTTTTTATT	152
	Nest 1,2	CRT -29.268_R	ATTTATTCATTCCTTTTTGT	
	Nest 2	CRT -29.268_F2	FAM-TATGTACCTCAGTAGACC	
-10.833kb	Nest 1	CRT -10.833_F1	TGCGTAAATTTTGATGTAAT	182
	Nest 1,2	CRT -10.833_R	CCCCTTCAAAAAGGAAATAACAC	
	Nest 2	CRT -10.833_F2	FAM-AAGAATGAAAGTATTTTTAGC	
-4.382kb	Nest 1	CRT -4.382_F1	GGTGTCAATTTTATTTTGTT	231
	Nest 1,2	CRT -4.382_R	ATACAATTTGGGGTGAAA	
	Nest 2	CRT -4.382_F2	FAM-GTCAATTTTATTTTGTTTCT	
-2.814kb	Nest 1	CRT -2.814_F1	AATTTCTACTAGTATCATAAACAA	184
	Nest 1,2	CRT -2.814_R	AAATCGAATTTATTTTATCG	
	Nest 2	CRT -2.814_F2	FAM-TTAAAAGCACCTTATTCATT	
0.59kb	Nest 1	CRT 0.59_F1	TAGAAATGAGAAGAAGCAAA	163
	Nest 1,2	CRT 0.59_R	ACCTATTTATCAAAACACCA	
	Nest 2	CRT 0.59_F2	FAM-AATTCATACTGTGTCAAAGG	
10.389kb	Nest 1	CRT 10.389_F1	GCTCACATCATTTCTAACAT	205
	Nest 1,2	CRT 10.389_R	ACATTTAAGAAAAACCCATT	
	Nest 2	CRT 10.389_F2	FAM-CGGTATGATTATAATTTGAGA	

23.576kb	Nest 1	CRT 23.576_F1	TTAATCCATACTGCAAAAAT	190
	Nest 1,2	CRT 23.576_R	TAAATGGAAAGGAGTTTGTA	
	Nest 2	CRT 23.576_F2	FAM-ATCCATACTGCAAAAATAAA	
39.576kb	Nest 1	CRT 39.576_F1	AAACACAATCAAATAAAGGA	201
	Nest 1,2	CRT 39.576_R	TTTTGTTTGGTACATTTTTC	
	Nest 2	CRT 39.576_F2	FAM-CACAATCAAATAAAGGAAAG	
78.942kb	Nest 1	CRT 78.942_F1	AATTTGCTACATCCACATT	107
	Nest 1,2	CRT 78.942_R	TGTTTAATGGAATAACAAAAA	
	Nest 2	CRT 78.942_F2	FAM-TGTGTTTGTGTATAGACATTTT	
103.089kb	Nest 1	CRT 103.089kb_F1	CCATACCATTAATTTGGTT	152
	Nest 1,2	CRT 103.089kb_R	AAAATAACTGTTTTCTTTCT	
	Nest 2	CRT 103.089kb_F2	6FAM-TCAGAAATGGTTACGTAACC	
130.883kb	Nest 1	CRT 130.883kb_F1	ATACACTGAACAATCTTTAT	121
	Nest 1,2	CRT 130.883kb_R	AGATTGGACGAAAAATATCGA	
	Nest 2	CRT 130.883kb_F2	6FAM-ATACCCTTGGTTACACTCAAA	
154.062kb	Nest 1	CRT 154.062kb_F1	AAAGAACGACAAGAAGTAAA	159
	Nest 1,2	CRT 154.062kb_R	AAAAATAGATAACTAACAATATGC	
	Nest 2	CRT 154.062kb_F2	6FAM-ACGACAAGAAGTAAAACAAA	
192.909kb	Nest 1	CRT 192.909kb_F1	GCTCCATTTAATCGAAACT	150
	Nest 1,2	CRT 192.909kb_R	TTTTGGTTATATTACTTGCAT	
	Nest 2	CRT 192.909kb_F2	6FAM-TCGAAACTAATGAAAAATTG	
315.305kb	Nest 1	CRT 315.305kb_F1	TCCCTTCTAAAAAGTTTACAT	221
	Nest 1,2	CRT 315.305kb_R	ACTGTTCAGGTAAATACACG	
	Nest 2	CRT 315.305kb_F2	HEX-AAACAAAAAGGTACACACAC	
468.503kb	Nest 1	CRT 468.503kb_F1	TGATATTTTAAGGAATCGAC	202
	Nest 1,2	CRT 468.503kb_R	CCGTTTATATATTCAGCTTG	
	Nest 2	CRT 468.503kb_F2	HEX-TTGTAACATTGTAATGTTGT	
