# Supplementary material for: Changing genetic profiles of Plasmodium falciparum piperaquine resistance in Southeast Asia over 25 years
Source: Antimicrob Agents Chemother. 2026 Feb 17;70(4):e01117-25. doi: 10.1128/aac.01117-25 (PMC13041312; doi:10.1128/aac.01117-25)
Supplement: File S2 — Prevalence of Pfcrt mutations associated with piperaquine resistance across study sites and countries by year. [file aac.01117-25-s0002.pdf]

Supplementary Table: Prevalence of *Pfcrt* mutations associated with piperaquine resistance across study sites and countries by year.

Study sites	Study years	Number of Mutation specimens									Total
		Wild-type	T93S	H97L	H97Y	F145I	I218F	M343I	M343L	G353V	
Battambang	2015	4	0	0	0	0	0	0	0	3	7
Battambang	2016	1	0	0	0	0	0	0	0	10	11
Pailin	2007	6	0	0	0	0	0	0	0	0	6
Pailin	2008	24	0	0	0	0	0	0	0	0	24
Pailin	2011	5	0	0	0	0	1	0	1	1	8
Pailin	2012	4	0	0	1	0	0	0	0	0	5
Pailin	2015	4	1	0	2	0	1	1	0	2	11
Pailin	2016	11	1	0	0	0	1	1	0	1	15
Pailin	2017	0	2	0	1	0	0	0	0	1	4
Pursat	2016	4	0	0	7	0	1	2	0	3	17
Pursat	2017	0	0	0	11	0	0	0	0	2	13
Pursat	2019	21	0	14	32	0	3	0	0	2	72
Pursat	2020	0	0	0	1	0	0	0	0	1	2
Rattanakiri	2016	9	0	0	0	3	0	0	0	0	12
Rattanakiri	2017	2	3	0	0	0	3	0	0	0	8
Rattanakiri	2018	3	1	0	2	0	0	0	0	0	6
Stung Treng	2018	70	13	0	1	4	6	1	0	0	95
Stung Treng	2019	65	10	0	1	3	5	0	0	0	84
Stung Treng	2020	0	0	0	1	0	0	0	0	0	1
Attapeu	2011	21	0	0	0	0	0	0	0	0	21
Attapeu	2014	4	0	0	0	0	0	0	0	0	4
Attapeu	2018	18	4	0	2	0	0	0	0	0	24
Champasak	2013	8	0	2	0	0	0	0	0	0	10
Champasak	2018	18	9	0	0	2	1	0	0	0	30
Salavan	2013	9	0	2	0	0	0	0	0	0	11
Salavan	2014	5	0	1	0	0	0	0	0	0	6
Salavan	2018	16	0	0	0	0	0	0	0	0	16
Savannakhet	2003	9	0	0	0	0	0	0	0	0	9
Savannakhet	2010	47	0	0	0	0	0	0	0	0	47
Savannakhet	2013	9	0	0	0	0	0	0	0	0	9
Savannakhet	2014	11	0	0	0	0	0	0	0	0	11
Sekong	2013	0	0	2	0	0	0	0	0	0	2
Sekong	2014	15	0	2	0	0	0	0	0	0	17
Sekong	2017	15	0	0	0	0	0	0	0	0	15
Kayin	2015	9	0	0	0	0	0	0	0	0	9
Kayin	2016	3	0	0	0	0	0	0	0	0	3
Kayin	2017	17	0	0	0	0	0	0	0	0	17
Kayin	2023	27	0	0	0	0	0	0	0	0	27
Srisaket	2015	1	0	0	0	1	5	0	0	1	8
Srisaket	2016	0	0	0	0	1	1	0	0	1	3
Srisaket	2017	0	0	0	2	3	3	0	0	4	12
Tak	1995	5	0	0	0	0	4	0	0	1	10
Tak	2013	7	0	0	0	0	0	0	0	0	7
Tak	2014	4	0	0	0	0	0	0	0	0	4
Tak	2015	5	0	0	0	0	0	0	0	0	5
Tak	2016	18	0	0	0	0	0	0	0	0	18
Ubon	2014	1	1	0	0	0	0	0	0	0	2
Ubon	2015	5	0	0	0	0	2	0	0	0	7
Ubon	2016	0	1	0	0	0	0	0	0	0	1
Ubon	2017	3	1	0	0	0	0	0	0	1	5
Ubon	2018	0	5	0	0	1	0	0	0	2	8
Yala	2016	17	0	0	0	0	0	0	0	0	17
Binh Phuoc	2011	13	0	0	0	0	0	0	0	0	13
Binh Phuoc	2012	12	0	0	0	0	0	0	0	0	12
Binh Phuoc	2016	4	4	0	0	0	5	0	0	0	13
Binh Phuoc	2017	0	8	0	0	3	2	1	0	0	14
Binh Phuoc	2018	3	5	0	2	5	2	0	0	0	17
Binh Phuoc	2019	1	4	0	0	11	6	0	0	0	22
Khanh Hoa	2018	4	0	0	0	0	1	0	0	0	5
Khanh Hoa	2019	5	0	0	0	0	1	0	0	0	6
**Total**		602	73	23	66	37	54	6	1	36	898
