# Supplementary material for: Changing genetic profiles of Plasmodium falciparum piperaquine resistance in Southeast Asia over 25 years
Source: Antimicrob Agents Chemother. 2026 Feb 17;70(4):e01117-25. doi: 10.1128/aac.01117-25 (PMC13041312; doi:10.1128/aac.01117-25)
Supplement: File S4 — Copy number variation (CNV) of pfplasmepsin2 in Cambodia, Thailand, and Vietnam. [file aac.01117-25-s0004.pdf]

Supplementary figure: Copy Number Variation (CNV) of *Pfplasmepsin2* in Cambodia, Thailand, and Vietnam

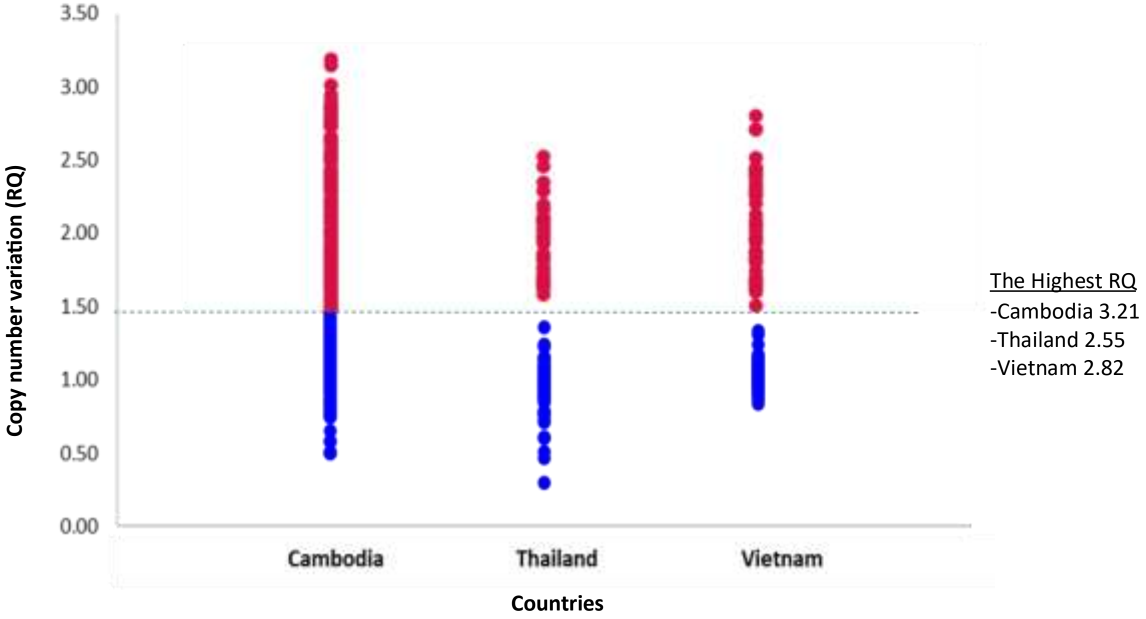

The figure displays the copy number variation (CNV) of *Pfplasmepsin2* across three Southeast Asian countries: Cambodia, Thailand, and Vietnam. These data are represented through a dot plot with a horizontal green dashed line at 1.5 marking a threshold. The y-axis represents the copy number variation (RQ). The range extends from 0 to approximately 3.50. Parasite isolates from Cambodia show the highest maximum RQ value at 3.21. Parasite isolates from Vietnam follow with a maximum RQ of 2.82. The parasite isolates from Thailand demonstrate the lowest maximum among the three at 2.55.
