# Supplementary material for: Changing genetic profiles of Plasmodium falciparum piperaquine resistance in Southeast Asia over 25 years
Source: Antimicrob Agents Chemother. 2026 Feb 17;70(4):e01117-25. doi: 10.1128/aac.01117-25 (PMC13041312; doi:10.1128/aac.01117-25)
Supplement: File S6 — Prevalence of Pfcrt haplotypes associated with chloroquine resistance. [file aac.01117-25-s0006.pdf]

Supplementary Table: Prevalence of *Pfcrt* haplotypes associated with Chloroquine resistance.

No	Countries	Study sites	Study Year	Number of Haplotypes					Total
				CVI/MET/K	CVIDT	CVIE/DT	CVIET	CVMNK	
1	Cambodia	Battambang	2015	0	0	0	7	0	7
2	Cambodia	Battambang	2016	0	0	0	11	0	11
3	Cambodia	Pailin	2007	0	0	0	6	0	6
4	Cambodia	Pailin	2008	0	0	1	23	0	24
5	Cambodia	Pailin	2011	0	0	0	8	0	8
6	Cambodia	Pailin	2012	0	0	0	5	0	5
7	Cambodia	Pailin	2015	0	0	0	11	0	11
8	Cambodia	Pailin	2016	0	0	0	14	1	15
9	Cambodia	Pailin	2017	0	0	0	4	0	4
10	Cambodia	Pursat	2016	0	0	0	17	0	17
11	Cambodia	Pursat	2017	0	0	0	13	0	13
12	Cambodia	Pursat	2019	0	0	0	72	0	72
13	Cambodia	Pursat	2020	0	0	0	2	0	2
14	Cambodia	Rattanakiri	2016	0	0	0	12	0	12
15	Cambodia	Rattanakiri	2017	0	0	0	8	0	8
16	Cambodia	Rattanakiri	2018	0	0	0	6	0	6
17	Cambodia	Stung Treng	2018	0	19	4	72	0	95
18	Cambodia	Stung Treng	2019	0	21	0	63	0	84
19	Cambodia	Stung Treng	2020	0	0	0	1	0	1
20	Lao PDR	Attapeu	2011	0	3	1	17	0	21
21	Lao PDR	Attapeu	2014	0	0	0	4	0	4
22	Lao PDR	Attapeu	2018	0	1	1	22	0	24
23	Lao PDR	Champasak	2013	0	1	0	9	0	10
24	Lao PDR	Champasak	2018	0	1	1	28	0	30
25	Lao PDR	Salavan	2013	0	4	0	7	0	11
26	Lao PDR	Salavan	2014	0	1	0	5	0	6
27	Lao PDR	Salavan	2018	0	6	0	9	1	16
28	Lao PDR	Savannakhet	2003	0	3	0	4	2	9
29	Lao PDR	Savannakhet	2010	0	13	0	22	12	47
30	Lao PDR	Savannakhet	2013	0	0	0	9	0	9
31	Lao PDR	Savannakhet	2014	0	0	0	11	0	11
32	Lao PDR	Sekong	2013	0	0	0	2	0	2
33	Lao PDR	Sekong	2014	0	0	0	17	0	17
34	Lao PDR	Sekong	2017	0	1	0	14	0	15
35	Myanmar	Kayin	2015	0	0	0	9	0	9
36	Myanmar	Kayin	2016	0	0	0	2	1	3
37	Myanmar	Kayin	2017	0	0	0	16	1	17
38	Myanmar	Kayin	2023	0	0	0	27	0	27
39	Thailand	Srisaket	2015	0	0	0	8	0	8
40	Thailand	Srisaket	2016	0	0	0	3	0	3
41	Thailand	Srisaket	2017	0	0	0	12	0	12
42	Thailand	Tak	1995	0	0	0	10	0	10
43	Thailand	Tak	2013	0	0	0	7	0	7
44	Thailand	Tak	2014	0	0	0	4	0	4
45	Thailand	Tak	2015	0	0	0	5	0	5
46	Thailand	Tak	2016	0	1	1	12	4	18
47	Thailand	Ubon	2014	0	0	0	2	0	2

48	Thailand	Ubon	2015	0	0	0	7	0	7
49	Thailand	Ubon	2016	0	0	0	1	0	1
50	Thailand	Ubon	2017	0	0	0	5	0	5
51	Thailand	Ubon	2018	0	0	0	8	0	8
52	Thailand	Yala	2016	0	0	0	17	0	17
53	Vietnam	Binh Phuoc	2011	1	5	3	3	1	13
54	Vietnam	Binh Phuoc	2012	0	6	1	4	1	12
55	Vietnam	Binh Phuoc	2016	0	0	0	13	0	13
56	Vietnam	Binh Phuoc	2017	0	0	0	14	0	14
57	Vietnam	Binh Phuoc	2018	0	0	0	17	0	17
58	Vietnam	Binh Phuoc	2019	0	0	0	22	0	22
59	Vietnam	Khanh Hoa	2018	0	4	0	1	0	5
60	Vietnam	Khanh Hoa	2019	0	2	0	3	1	6
**Total**				1	92	13	767	25	898
